# Sound Decision Making in Uncertain Times: Can Systems Modelling Be Useful for Informing Policy and Planning for Suicide Prevention?

**DOI:** 10.3390/ijerph19031468

**Published:** 2022-01-27

**Authors:** Jo-An Occhipinti, Danya Rose, Adam Skinner, Daniel Rock, Yun Ju C. Song, Ante Prodan, Sebastian Rosenberg, Louise Freebairn, Catherine Vacher, Ian B. Hickie

**Affiliations:** 1 Brain and Mind Centre, Faculty of Medicine and Health, University of Sydney, Sydney, NSW 2006, Australia; danya.rose@sydney.edu.au (D.R.); adam.skinner@sydney.edu.au (A.S.); yun.song@sydney.edu.au (Y.J.C.S.); a.prodan@westernsydney.edu.au (A.P.); sebastian.rosenberg@sydney.edu.au (S.R.); louise.freebairn@sydney.edu.au (L.F.); catherine.vacher@sydney.edu.au (C.V.); ian.hickie@sydney.edu.au (I.B.H.); 2 Computer Simulation & Advanced Research Technologies (CSART), Sydney, NSW 2021, Australia; 3 Medical School, University of Western Australia, Perth, WA 6009, Australia; daniel.rock@uwa.edu.au; 4 WA Primary Health Alliance, Perth, WA 6008, Australia; 5 School of Computer, Data and Mathematical Sciences, Western Sydney University, Penrith, NSW 2751, Australia; 6 St Vincent’s Clinical School, University of New South Wales, Sydney, NSW 2052, Australia

**Keywords:** suicide prevention, strategic planning, decision analysis, systems modelling, simulation, mental health

## Abstract

The COVID-19 pandemic demonstrated the significant value of systems modelling in supporting proactive and effective public health decision making despite the complexities and uncertainties that characterise an evolving crisis. The same approach is possible in the field of mental health. However, a commonly levelled (but misguided) criticism prevents systems modelling from being more routinely adopted, namely, that the presence of uncertainty around key model input parameters renders a model useless. This study explored whether radically different simulated trajectories of suicide would result in different advice to decision makers regarding the optimal strategy to mitigate the impacts of the pandemic on mental health. Using an existing system dynamics model developed in August 2020 for a regional catchment of Western Australia, four scenarios were simulated to model the possible effect of the COVID-19 pandemic on levels of psychological distress. The scenarios produced a range of projected impacts on suicide deaths, ranging from a relatively small to a dramatic increase. Discordance in the sets of best-performing intervention scenarios across the divergent COVID-mental health trajectories was assessed by comparing differences in projected numbers of suicides between the baseline scenario and each of 286 possible intervention scenarios calculated for two time horizons; 2026 and 2041. The best performing intervention combinations over the period 2021–2041 (i.e., post-suicide attempt assertive aftercare, community support programs to increase community connectedness, and technology enabled care coordination) were highly consistent across all four COVID-19 mental health trajectories, reducing suicide deaths by between 23.9–24.6% against the baseline. However, the ranking of best performing intervention combinations does alter depending on the time horizon under consideration due to non-linear intervention impacts. These findings suggest that systems models can retain value in informing robust decision making despite uncertainty in the trajectories of population mental health outcomes. It is recommended that the time horizon under consideration be sufficiently long to capture the full effects of interventions, and efforts should be made to achieve more timely tracking and access to key population mental health indicators to inform model refinements over time and reduce uncertainty in mental health policy and planning decisions.

## 1. Introduction

At the outset of the COVID-19 pandemic, systems models were rapidly deployed in many countries to estimate the likely trajectories of transmission, mortality, and health system burden, to determine the most impactful mitigation strategies, and to most effectively allocate limited resources [1,2,3]. The extent to which decision makers engaged with modelling and simulation to help inform proactive and timely actions to arrest virus transmission varied across nations. Countries, such as Australia and New Zealand, that engaged early and consistently with the modelling, avoided the significant adverse impacts on health system, economic, and social indicators seen elsewhere in the world. For example, Australia’s early mandated suppression strategies that were informed by the Doherty Institute’s original modelling [4] have since been estimated to have prevented tens of thousands of deaths from COVID-19 compared to delayed mandated suppression, and prevented ICU demands that would have been up to 40 times the capacity of the healthcare system, saving $13.5 billion in health care costs, and preventing substantial losses to the Australian economy compared to a strategy of unmitigated spread [5]. The pandemic has helped to highlight the significant value of systems models as decision support tools, providing the ability to test the likely impact of policy and planning scenarios (helping to understand what combination of strategies are needed, at what time, at what scale, and for how long), and informing proactive and effective action despite the complexity, uncertainties, and imperfect knowledge that characterise an evolving crisis [6]. Beyond its benefits for informing decision making, systems modelling has long been used to advance scientific understanding of the spread of human disease from the first compartmental model of smallpox described by Daniel Bernoulli in 1776, to the Nobel Prize winning dynamic transmission modelling of malaria developed by Ronald Ross in the early 20th Century [7].

In recent times there have been sustained calls for more routine use of the systems modelling approach in mental health research and decision making as a key strategy in addressing the disappointing progress on population mental health outcomes over decades and to inform mitigation of the social and economic impacts of the pandemic on mental health [6,8,9,10,11,12]. Evidence from systems modelling applications to answer questions related to mental health systems strengthening, system reform, and investments in the social determinants of mental health have elucidated a range of important insights. These insights include, (i) that more is not necessarily better, i.e., investing in programs and initiatives beyond the best performing combination can deliver little additional benefit [13]; (ii) that even evidence based interventions can fail to deliver impact or can potentially result in unintended consequences [14]; (iii) that health systems exhibit non-linear behaviour and threshold effects that have implications for system investment [15,16]; (iv) that some intervention combinations and system reforms have the potential to deliver synergistic effects, i.e., where the impact of key strategies combined is greater than the sum of their impact if implemented in isolation [14,17]; (v) that some social determinants of mental health can be more important than others [18]; (vi) that regional variation in population and health system characteristics modifies the impact of suicide prevention measures on local suicide rates [19]; and (vii) that there can be marked trade-offs between minimising different population mental health outcomes, which have significant implications for cross-agency planning when there are competing priorities [20]. This growing evidence suggests that the comprehensive, ‘evidence-based’ approach long promoted by the population health research community and embraced by public health planners lacks nuance, focus, and strategic sophistication. As a result of the ‘comprehensive’ approach, decades of national mental health and suicide prevention action plans have included a promiscuous array of programs and initiatives that have delivered disappointing impacts and created mental health systems that are difficult to navigate and lack continuity and coordination of care [11].

Despite the promise systems modelling presents to population mental health, a common misperception contributes to a resistance to engage with modelling and simulation in research, policy, and planning; that is, that the presence of parameter uncertainty can render such models useless. Uncertainty is indeed an important consideration for model credibility and the appropriate interpretation of modelling results. Sources of uncertainty can include a lack of data availability or quality, a lack of contextually relevant, generalisable research evidence, and/or a highly dynamic, evolving situation, such as a natural disaster or outbreak of a novel infectious disease. However, even in the early stages of the pandemic when there was sparse data on key input parameters of coronavirus transmission models, these models remained valuable to decision making. The same is possible for decision making to improve population mental health modelling that benefits from decades of research and administrative data collection. However, in modelling the social and economic impacts of COVID-19 on mental health there remain uncertainties in parameters that would significantly influence the mental health trajectory over the next five years. Specifically, it is unclear to what extent the disruption, social dislocation, and financial hardships brought about by the pandemic will increase rates of psychological distress. In order to understand whether this uncertainty renders systems models inadequate for informing effective mitigation strategies, we used an existing regional mental health model to explore whether radically different simulated trajectories of an important mental health outcome (suicide deaths) would result in different advice to decision makers regarding the optimal mitigation strategy.

## 2. Materials and Methods

### 2.1. Context, Model Structure and Outputs

**Figure 1:**
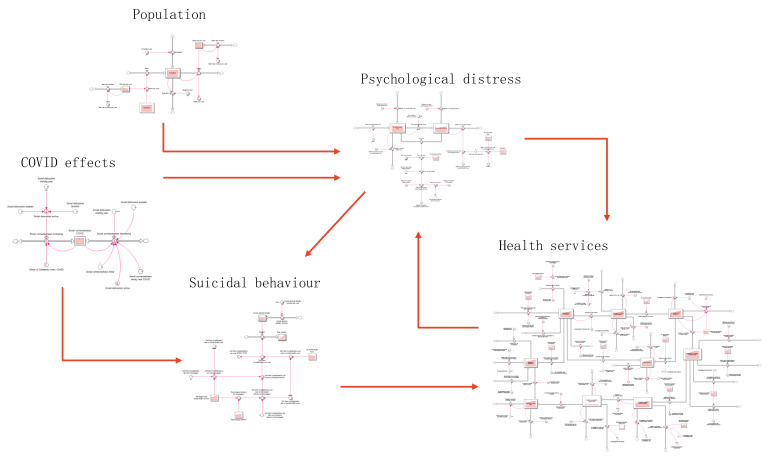
High-level map of the core system dynamics model showing the causal connections among model sectors. Single-headed arrows indicate unidirectional causal connections; bidirectional causal connections are shown as double-headed arrows.

**Figure 2:**
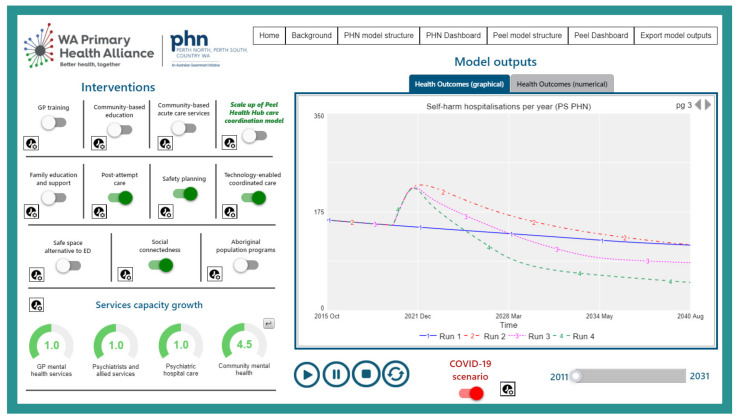
Interactive model interface.

This analysis was undertaken using an existing regional system dynamics model developed in August 2020 for the Perth South Primary Health Network (PHN) population catchment. Perth South PHN is a metropolitan region of Western Australia, covering 5069 square kilometres with an estimated resident population of 973,769 [21,22]. The system dynamics model developed was based on a similar model reported elsewhere [20] that was reviewed, re-parameterised, and verified in partnership with Perth South PHN collaborators to ensure that the model structure and assumptions were valid for the Perth South context. Briefly, the model includes: (1) a population component, capturing changes over time in population size resulting from births, migration, and mortality; (2) a psychological distress component that models flows of people to and from states of low or no psychological distress (Kessler 10 [K10 scores below 15), and moderate to very high psychological distress (K10 score 16−50); (3) a mental health services component that models the movement of psychologically distressed people through possible service pathways across the primary to tertiary service continuum involving (potentially) general practitioners (GPs), psychiatrists and allied mental health professionals (including psychologists, mental health nurses, social workers, etc.), psychiatric inpatient care, community mental health centres, and online services; (4) a suicidal behaviour component that captures self-harm hospitalisations and suicide deaths; and (5) a COVID-19 component that captures the impact of the pandemic and recession on social connectedness, unemployment, and psychological distress from 1 March 2020. The primary model output used for this analysis was the total (cumulative) numbers of suicide deaths. Figure 1 presents a high-level map of the system dynamics model showing the (causal) interconnections between the components and Figure 2 presents the interactive user interface of the model.

Parameter estimates and other numerical inputs were derived (where possible) from published research and available data or were estimated via constrained optimisation using historical time series data. Powell’s method [23] was employed to obtain the set of (optimal) parameter values, minimising the sum of the mean absolute percent error calculated for each time series separately (i.e., the mean of the absolute differences between the observed time series values and the corresponding model outputs, where each difference is expressed as a percentage of the observed value). The model broadly reproduces historic trends across a range of indicators, including the prevalence of psychological distress, mental health-related emergency department (ED) presentations, self-harm hospitalisations, suicide deaths, and service referrals, from 2011–2017/18. In addition to the ability to scale up or down mental health services capacity captured in the core structure of the model, a range of possible mental health and suicide prevention programs and initiatives were integrated into the model, including post-suicide attempt care, general practitioner training, community-based education programs, family psychoeducation and support, safety planning, safe space services (based on the UK’s Safe Haven café model), social connectedness programs, community-based acute care services, and technology enabled care coordination. Supplementary Materials (Figures S1–S15, Tables S1–S3) provides a detailed description of each of the model components, their interconnections, parameter inputs, and model validation graphs (Section 1 and Section 2), as well as intervention definitions and the research evidence used to inform default intervention parameter values (Section 3). Model construction and analysis were performed using Stella Architect version 1.9.4 [24].

### 2.2. Policy Testing and Sensitivity Analyses

The substantial adverse mental health impacts of social dislocation and job loss resulting from the continuing COVID-19 pandemic [25,26,27] were modelled primarily as an increase in psychological distress incidence from 1 March 2020 that declines gradually until the end of the simulation period. The scale (denoted by CES, i.e., COVID-19 Effect Scale) and duration (denoted by CED, i.e., COVID-19 Effect Duration) of the COVID-19 effect on psychological distress are the key uncertain parameters that influence the trajectory of the primary outcome (suicide deaths) that were determined through preliminary sensitivity analysis. Parameters controlling the modelled effect of the COVID-19 pandemic on psychological distress onset are detailed in Table S1. Specifically, we considered four scenarios of the COVID-19 effect on psychological distress that resulted in a range of projected impacts on rates of suicide, from very little to dramatic:

- Scenario A: short duration (CED = 0.5 years) and low impact (CES = 0.11)—lowest projected increase in suicides
- Scenario B: short duration (CED = 0.5 years) and high impact (CES = 0.33),
- Scenario C: long duration (CED = 1.5 years) and low impact (CES = 0.11),
- Scenario D: long duration (CED = 1.5 years) and high impact (CES = 0.33)—highest projected increase in suicides

Determining the optimal combination of interventions: The effectiveness of different combinations of interventions were explored across a range of possible estimates of the scale and duration of the adverse COVID-19 effect on psychological distress to see whether the best performing set of three interventions for reducing suicide deaths were consistent or inconsistent across the alternative trajectories. Our choice of intervention set size of three reflects the fact that suicide prevention programs are generally implemented within resource-constrained settings, where only a limited number of interventions can be supported and implemented simultaneously. Potential discordance in the best-performing intervention scenarios across the four COVID-mental health scenarios (A–D) was assessed by examining reductions in the total (cumulative) numbers of suicides under all possible combinations of three interventions selected from the 13 programs, services and initiatives modelled. Differences in projected numbers of suicides between the baseline scenario and each optimal intervention scenario were calculated using two different time horizons; the period 2021–2026, and the period 2021–2041.

Sensitivity analyses were performed to assess the impact of uncertainty in estimates of the direct effects of each intervention and forecasted growth in services capacity (i.e., GP mental health services, psychiatrists and allied services, community mental health services, and psychiatric hospital care) on the simulation results. We used Latin hypercube sampling to draw 100 sets of values for the selected model parameters from a uniform joint distribution spanning ±20% of the default values. The resulting 95% intervals generated for the projected impact of each intervention combination provide a measure of the effect of uncertainty, but should not be interpreted as confidence intervals.

## 3. Results

**Figure 3:**
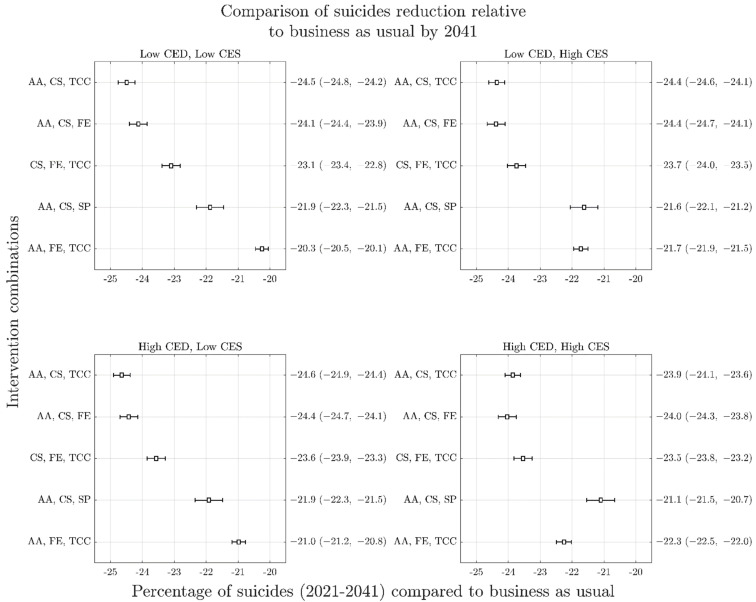
Forest plots arising from sensitivity analyses of reduction in cumulative suicide deaths (2021–2041) as a result of top performing intervention combinations across the four COVID-19 mental health scenarios. Panels represent different COVID-19 scenarios: top left, short duration and low impact (Scenario A); top right, short duration and high impact (Scenario B); bottom left, long duration and low impact (Scenario C); bottom right; long duration and high impact (Scenario D). The *y*-axis of each panel presents the mean percent reduction in cumulative suicides against the baseline (business as usual) for each intervention combination with uncertainty intervals in brackets. Overlapping 95% intervals indicate possible ambiguity of rankings within each COVID-19 mental health scenario, relating to the uncertainty in intervention effect sizes and services capacity growth rates. Similarity of possible rankings between scenarios is indicative that uncertainty about the effects of COVID-19 on mental health do not change recommendations about optimal intervention investments. AA is post-suicide attempt aftercare; CS is community support programs to increase community connectedness; SP is safety planning; FE is family education and support; TCC is technology-enabled care coordination.

Table 1 provides the percent increase in cumulative suicide deaths over the period 2020–2041 (with uncertainty intervals) for the four COVID-mental health scenarios. These increases are measured against a scenario of the pandemic having not occurred. Figure 3 provides the percent reduction in cumulative suicides against the baseline (business as usual) with uncertainty intervals for the five best performing intervention combinations for each of the four COVID-19 mental health scenarios (A–D) over the period 2021–2041. These results demonstrate that the top two best performing intervention combinations (i.e., (i) post-suicide attempt assertive aftercare, community support programs to increase community connectedness, and technology enabled care coordination; (ii) post-suicide attempt assertive aftercare, community support programs to increase community connectedness and family education and support) delivered impacts that were highly consistent across all four possible COVID-19 mental health trajectories, reducing suicide deaths by between 23.9–24.6% against the baseline.

**Figure 4:**
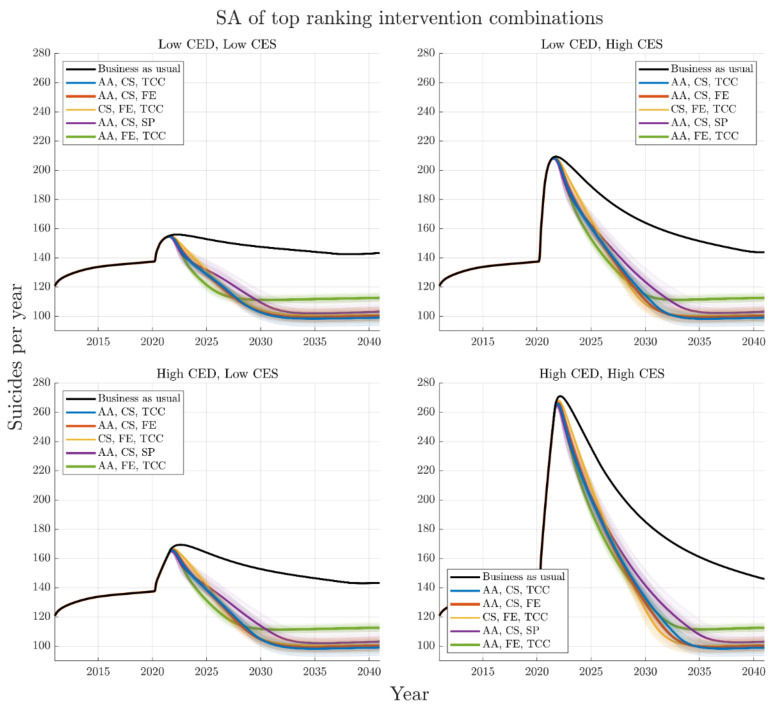
Trajectories for the best performing intervention combinations in reducing suicides deaths over the period 2021–2041 for the four different COVID-19 mental health scenarios: top left, short duration and low impact (Scenario A); top right, short duration and high impact (Scenario B); bottom left, long duration and low impact (Scenario C); bottom right; long duration and high impact (Scenario D). Default parameters are chosen for each intervention. The top three ranking sets of interventions are consistent across COVID-19 scenarios; however, the fourth top intervention combination differs depending on CES. The thick black curve indicates the business-as-usual case, the coloured curves indicate the top performing intervention combinations for reducing cumulative suicides from 2021–2041. Distribution means are indicated with a heavy line, and span of individual trajectories from the 100 runs of the sensitivity analysis are presented. AA is post-suicide attempt aftercare; CS is community support programs to increase community connectedness; SP is safety planning; FE is family education and support; TCC is technology-enabled care coordination.

**Figure 5:**
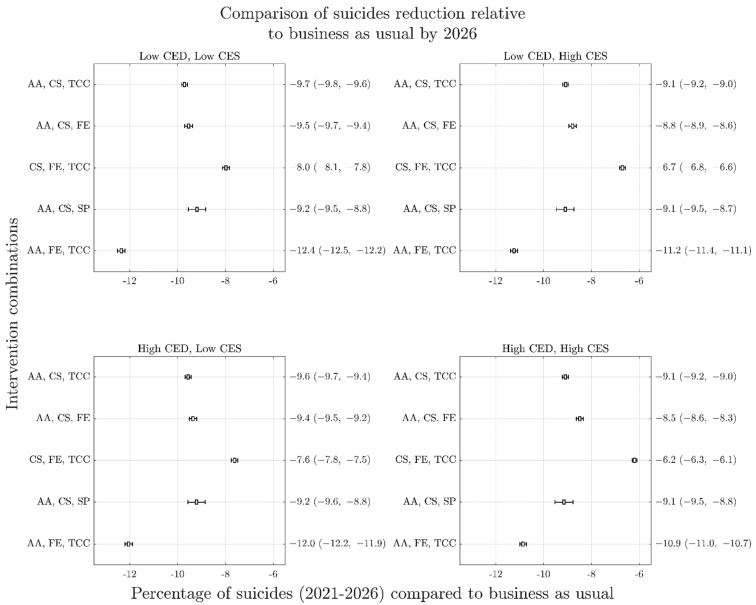
Forest plots similar to Figure 3 for percent reduction in cumulative suicides over the period 2021–2026 as a result of top performing intervention combinations. Note substantially different performance rankings from Figure 3 but similarity of rankings across COVID-19 mental health scenarios. Panels represent different COVID-19 scenarios (A–D) as per previous figures. AA is post-suicide attempt aftercare; CS is community support programs to increase community connectedness; SP is safety planning; FE is family education and support; TCC is technology-enabled care coordination.

Figure 4 shows time series graphs of the best performing combinations across the four COVID-19 mental health scenarios (A–D), demonstrating their non-linear impacts over time. Results of the analysis of best performing interventions for different time horizons are presented in Figure 3 (with a 2041-time horizon) and Figure 5 (with a 2026-time horizon). These results demonstrate that for each COVID-19 mental health trajectory, the ranking of best performing intervention combinations changes depending on the time horizon under consideration. For example, the best performing combination of interventions for the 2041-time horizon under the most conservative COVID-19 mental health scenario (i.e., Scenario A) includes post-suicide attempt aftercare, community support programs to increase community connectedness, and technology enabled care coordination, delivering a 24.5% (95% interval, 24.2–24.8%) reduction in suicide deaths against the baseline. However, the best performing combination of interventions for the 2026-time horizon under Scenario A includes post-suicide attempt aftercare, family education and support, and technology-enabled care coordination, delivering a 12.4% (95% interval, 12.2–12.5%) reduction in suicide deaths against the baseline. However, the rankings of best performing intervention combinations are largely consistent between COVID-19 scenarios (A–D) at any given horizon.

**Figure 6:**
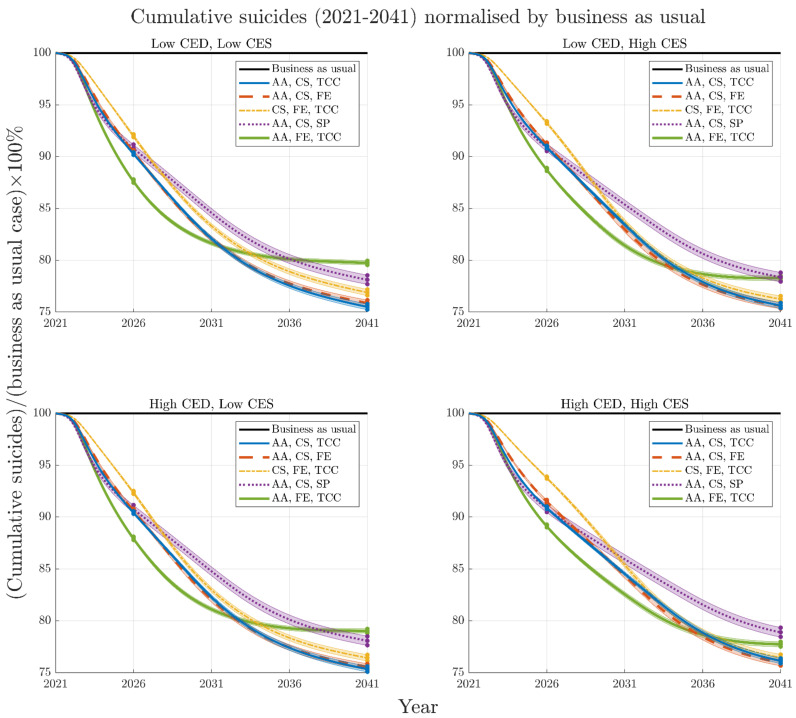
Mean and 95% intervals of cumulative suicides for each top performing combination of interventions (normalised by respective business as usual cases) for the four COVID-19 mental health scenarios (A–D) from 2021–2041. Time slices illustrated in Figure 3 and Figure 5 are noted at 2026 and 2041. Note that while different combinations of interventions change rankings over time, the rankings (including 95% intervals) remain similar regardless of the severity or duration of the COVID-19 mental health scenario.

The difference in rankings due to time horizon is a result of some intervention combinations acting quickly to reduce suicide deaths while others are slower to realise their full impact but have amplifying effects over time. This is highlighted in Figure 6, which presents the mean and 95% intervals of cumulative suicides for each of the five top performing combinations of interventions normalised by respective business as usual cases. The time slices at 2026 in Figure 6 indicate the best performing combination to consist of post-suicide attempt aftercare, family education, and technology-enabled care coordination, however, this is no longer the case by 2041. Analyses, in which optimal sets of four interventions are selected from the 12 modelled interventions, yield results qualitatively similar to those in Figure 3 (Figure S15).

## 4. Discussion

This study aimed to determine whether the presence of input parameter uncertainty pertaining to the impacts of the pandemic on the trajectory of suicide deaths renders systems models inadequate for informing best mitigation strategies. The findings showed that despite simulating four vastly different scenarios relating to the potential impact of the pandemic on rates of moderate to very high psychological distress and hence the trajectory of suicide deaths, the best performing combinations of three interventions selected from the 13 interventions modelled remained highly consistent across the alternative COVID-19 mental health trajectories. For the Perth South PHN population catchment, the best performing intervention combinations projected for the period 2021–2041 included post-suicide attempt assertive aftercare, community support programs to increase community connectedness, technology enabled care coordination, and family education and support. While a broader range of programs, services, and initiatives not examined in the current study may offer value, and while different combinations may perform best in different regions, these findings suggest that systems models offer value in guiding investments in suicide prevention even in the presence of significant uncertainty in the COVID-19 mental health trajectory.

Systems modelling-based decision analysis provides a systematic, robust, and objective basis for determining the most effective combination, scale, targeting, timing and duration of interventions needed to deliver impact on key population health outcomes; advantages that have been increasingly recognised in recent times with their use in responding to both the physical and mental health threats posed by the pandemic [4,10,28,29]. However, when the use of such models is for the purpose of estimating future burden of disease, healthcare costs, or surge capacity planning in mental health care systems rather than strategic decision analysis, greater precision around the likely future trajectory becomes far more important. Therefore, strengthening the mental health data ecosystem in Australia to support systems modelling, and establishing mechanisms for continuous feedback between real world and modelled systems will be important for reducing uncertainty around projected trajectories of population mental health outcomes and estimates of the resources needed to change those trajectories.

Despite the improvements yet to be made in strengthening population mental health data and compiling further empirical evidence on the impact of the pandemic on mental health and suicide outcomes, at what cost do we wait for greater certainty before engaging with decision analytic tools grounded in complexity science that can provide insights into effective strategic actions? Concerns about model uncertainty need to be balanced against the known limitations of existing approaches to mental health planning. Investments and actions that rely on issues to first be realised and signalled in the data does not provide systems with the capacity to understand and proactively address shifting contemporary mental health needs in communities [6]. The pandemic has demonstrated how unfit for purpose these traditional approaches to mental health planning are. Even in the presence of uncertainty (and because of it), systems modelling approaches provide important new planning infrastructure in mental health.

Another key finding of this study was the importance of the time horizon in estimating both the optimal combination of interventions to inform a strategic response, and the impact that optimal combination is likely to have. Even under the most conservative scenario of the trajectory of suicide deaths, this study showed that the best performing combination of interventions for the 2041-time horizon delivered double the percent reduction in cumulative suicide deaths against the baseline than the 2026-time horizon did due to non-linear intervention impacts. This has important implications for decision making that seeks to make the best use of limited public health resources but represents a current challenge in the context of short funding cycles and the political desire to provide ‘instant solutions’ [30,31]. While time horizons that are too long are likely to be perceived by decision makers as impractical, and in themselves represent an additional source of uncertainty, good practice guidelines in dynamic modelling and simulation recommend that the time horizon be sufficiently long to capture all the effects of an intervention [32]. Figure 3 demonstrates a plateauing of intervention impacts well beyond the 2026-time horizon, suggesting that this shorter time horizon would be inadequate for an analysis of the optimal intervention combination. The unique value of systems modelling methods in accounting for intervention scale up, time to full effect, and non-linear intervention impacts have previously been highlighted and this knowledge can assist in supporting longer term policy and program planning and decision making [33].

## 5. Limitations

The key limitation of this work is the lack of examination of the impact of the structural uncertainty of the model on findings. Structural uncertainty relates to the possibility that multiple alternative representations of a complex system could reproduce observed data, but give rise to divergent model behaviours and outputs [34]. The impact of the structural uncertainty of models (as opposed to parameter uncertainty) is often ignored due to it being very difficult to quantify, particularly for high dimensional models. While it has been proposed that a range of structural representations of a complex system that reproduce observed data be developed and the divergence in their forecasts examined [35,36], this is often unfeasible within the timeframe of a modelling project and may not necessarily adequately capture the extent of the uncertainty. However, participatory model building processes can contribute to improving the structural validity of models during their development, and particle filtering methods can contribute to reducing the impact of structural uncertainty as an ongoing process.

The model used for the current analysis was originally developed using a broad and inclusive participatory process involving stakeholders from state governments, health and social policy agencies, local councils, non-government organisations, the education sector, emergency services, research institutions, community groups, primary care providers, multidisciplinary researchers, indigenous representatives, and people with lived experience of suicide [20]. The model was further verified during the re-parameterisation processes with Perth South PHN collaborators. This process sought to reduce structural uncertainty by ensuring model structure and assumptions were, as far as possible, informed by the available empirical evidence and exposed to critique by those with diverse perspectives and knowledge of that system.

A model’s structure drives its dynamics [37]. Particle filtering is a machine learning (sequential Monte Carlo state inference and identification) method that uses new observational time series data to characterise and correct for uncertainty in model dynamics [38,39]. While widely employed in non-health fields, such as robotics, particle filtering is only more recently being applied in health, particularly to infectious disease models [39,40,41,42,43,44]. Similarly, mental health time series data (which itself can be noisy and offer little capacity for predicting future trajectories or the impacts of interventions) could be used to continuously reground dynamic models (which can provide accurate shorter-term projections but diverge from empirical patterns over the longer term) to mitigate the weaknesses of both and confer greater reliability in forward projections [45]. Particle filtering enables the recurrent updating of systems modelling-based decision support tools to ensure their ongoing usefulness and can offer reliable forecast capability even in the context of unanticipated events that lie outside of the scope of the model [45].

## 6. Conclusions

Achieving representation of a complex system with absolute certainty is impossible. As with much of science, seeking ‘truth’ is an ongoing process, where a theory stands because it enjoys shared confidence in its likelihood and has not yet been disproven; ‘likewise, one tests a system dynamics model against a diversity of empirical evidence, seeks disproofs, and develops confidence as the model withstands tests’ over time [37]. In the meantime, the COVID-19 pandemic has demonstrated the significant value of systems modelling and simulation in empowering governments that engaged with such tools to act proactively and effectively despite uncertainties and imperfect knowledge that characterised the evolving crisis. The findings of this study suggest that systems modelling informed decision making in population mental health has the potential to be robust even in the presence of significant variation in the simulated trajectory of suicide deaths that could arise due to parameter uncertainty. However, efforts should continue to be made to achieve more timely tracking and access to key population mental health indicators, including the prevalence of psychological distress and incidence of suicidal behaviour, to inform model refinements and reduce uncertainty in mental health policy and planning. In addition, efforts should be made to ensure that known sources of uncertainty are acknowledged, and further research should focus on improving methods to measure model uncertainty.

## Figures and Tables

**Table 1:** Percent increase in cumulative suicide deaths over the period 2020–2041 (with 95% intervals) for the four COVID-19 mental health scenarios.

Suicide Deaths	Scenario A	Scenario B	Scenario C	Scenario D
% increase compared to no pandemic	4.9	18.6	8.1	34.7
95% intervals *	4.5, 5.3	18.1, 19.1	7.7, 8.5	33.9, 35.5

* Uncertainty intervals presented are a measure of the impact of uncertainty of projected growth in services capacity on the simulation results and should not be interpreted as confidence intervals.

## Data Availability

Details of all data sources used for the analyses are provided throughout the Supplementary Materials, particularly in Tables S1–S3.

## Supplementary Materials

The following are available online at https://www.mdpi.com/article/10.3390/ijerph19031468/s1, Detailed model structure, assumptions, and parameters underpinning the system dynamics model. Figure S1: High-level map of the core system dynamics model showing the causal connections among model sectors. Single-headed arrows indicate unidirectional causal connections; bidirectional causal connections are shown as double-headed arrows. Figure S2: Structure of the population sector. Figure S3: Population estimate for the Perth South PHN (2011–2019) derived from the simulation model and from the Australian Bureau of Statistics (https://www.abs.gov.au/, accessed on 8 November 2021). See main text for detail on estimation of PHN population data from ABS SA3 data. Figure S4: Stock and flow structure of the psychological distress sector. Figure S5: Psychological distress prevalence estimate for the Perth South PHN, derived from the system dynamics model (red line, 2) and corresponding PHN and ABS National Health Survey data and WA Health and Wellbeing Surveillance System (blue line, 1). Figure S6: High-level map of the mental health services sector. Figure S7: Stock and flow structure of the help-seeking, general practitioner (GP) services, and online services components of the mental health services sector. Figure S8: Mental health services usage rates derived from the system dynamics model and from Medicare Benefits Schedule (MBS) data, data published by the Australian Institute of Health and Welfare (AIHW), and data available from Perth South PHN. Figure S9: Stock and flow structure of the psychiatrist and allied health services component of the mental health services sector. Figure S10: Stock and flow structure of the hospital services component of the mental health services sector. Figure S11 Stock and flow structure of the disengagement component of the mental health services sector. Figure S12: Structure of the suicidal behaviour sector. Figure S13 Self-harm hospitalisation and suicide death rate estimates derived from the system dynamics model and from Perth South PHN, ABS data (suicides) and the Australian Institute of Health and Welfare (2018) (self-harm hospitalisations). Figure S14: Shape of modelled impact of the continuing COVID-19 pandemic on psychological distress across the Perth South PHN catchment. The four COVID-19 scenarios vary the height and duration of the increase in distress onset against a baseline scenario (i.e., had the pandemic not occurred). Figure S15: Forest plots arising from sensitivity analyses of percent reduction in cumulative suicide deaths (2021–2041) as a result of top performing intervention combinations (set of four interventions) across the four COVID-mental health scenarios. Panels represent different COVID scenarios (A, B, C, D) as per Figure 3 in the main paper. Similar to the results reported in the paper, uncertainty about the effects of COVID on mental health do not change recommendations about optimal intervention selection. AA is post-suicide attempt aftercare; CS is community support programs to increase community connectedness; SP is safety planning; FE is family education and support; TCC is technology-enabled care coordination. Table S1: Parameters controlling the modelled effect of the COVID-19 pandemic on the incidence of psychological distress. Table S2: Numerical inputs and data sources. Inputs highlighted in red were varied in the sensitivity analyses (see Methods section of the paper). Table S3: Intervention definitions and parameter assumptions. Parameters determining the direct effects of each intervention can be modified via an interactive model interface, enabling users to assess the impact of parameter assumptions on model outputs.
